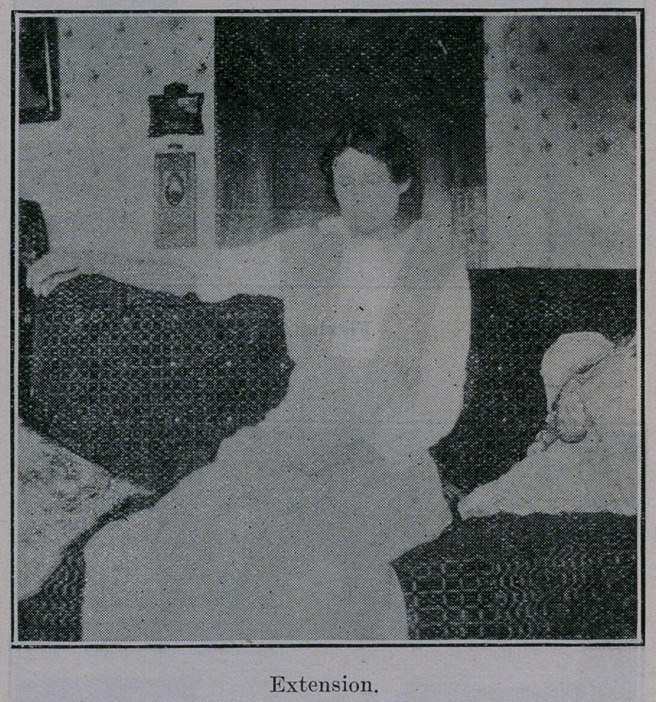# Dislocation of Elbow Joint and Flexed Ankylosis of the Knee Joint

**Published:** 1915-12

**Authors:** Joe Gilbert

**Affiliations:** Austin, Texas


### Dislocation of Elbow Joint and Flexed Ankylosis of Knee Joint.

BY JOE GILBERT, M. D., F. A. C. S., AUSTIN, TEXAS.

Miss S., 30 years of age; school teacher by occupation. Fell off a horse, dislocating right elbow joint. Attending physician placed arm in some form of sling or splint, and told patient it

Dislocation Radius and Ulna.

was a sprain. She came to Austin three months later with an extended, ankylosed arm.

Underwent treatment by physician here, who attempted reducing under an anesthetic, with no beneficial results.

Flexion.

Flexion and Rotation.

Came to me with a joint much swollen and inflamed as a result of manipulation. On account of this inflammation my operation was postponed for four or five weeks.

*Operation.*—Incision four inches long over joint externally; loosened up tissues as much as possible; tried to reduce dislocation. Found it impossible, except by cutting off head of radius and part of olecranon process. By doing this we reduced the dislocation. Then dissected fat and fascia which cover flexor muscles and placed between the bones, as is done by John B. Murphy.

Extension.

The arm was put up in flexed and partially supine position, with anterior plaster splint.

We began slight passive movement about seventh day. This was done in summer, and in the fall (one year ago) she had regained use of her arm so well as to resume teaching without inconvenience.

Noticing cuts, you will see that she now has all movements, supination, pronation, flexion and almost complete extension.

This case is illustrative of some points well worth remembering:

1. Never be satified with a diagnosis of sprain or torn ligaments until an X-ray is taken.

2. Don't leave a patient with a stiff and partially useless joint without trying surgical procedures—strict, aseptic, surgical methods in a well-regulated hospital.

Another interesting case of a young girl with a flexed ankylosed knee-joint (of two years' duration) came under my observation and treatment little over a year ago. In this case the patella was ankylosed to the lower end of the femur, and had to be removed. Ligamentum patella torn completely from its insertion on the tibia. (This was done under an anesthetic by a professional masseur and left in that condition for two years, patient walking on crutches.) Cut down on joint as is usual, removed patella, broke up ankylosis, transplanted fat and fascia from contiguous muscles in front of joints and stitched fat and fascia between bones, as in preceding case.

Unfortunately, we misplaced the X-ray photos in this case. Patient is now using limb with very slight inconvenience. Instead of a flexed, ankylosed knee, she now has a straight limb, with partial movement in the joint.

## Figures and Tables

**Figure f1:**
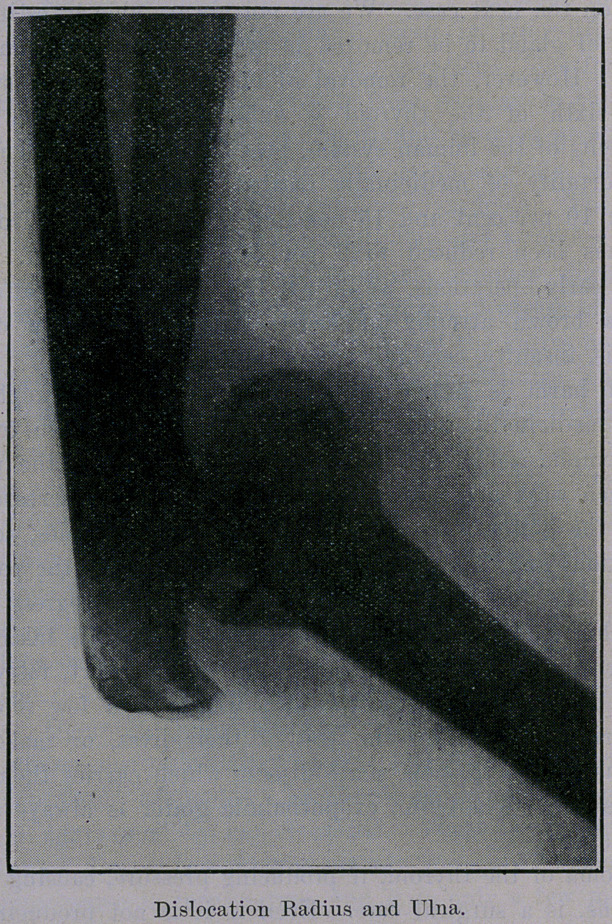


**Figure f2:**
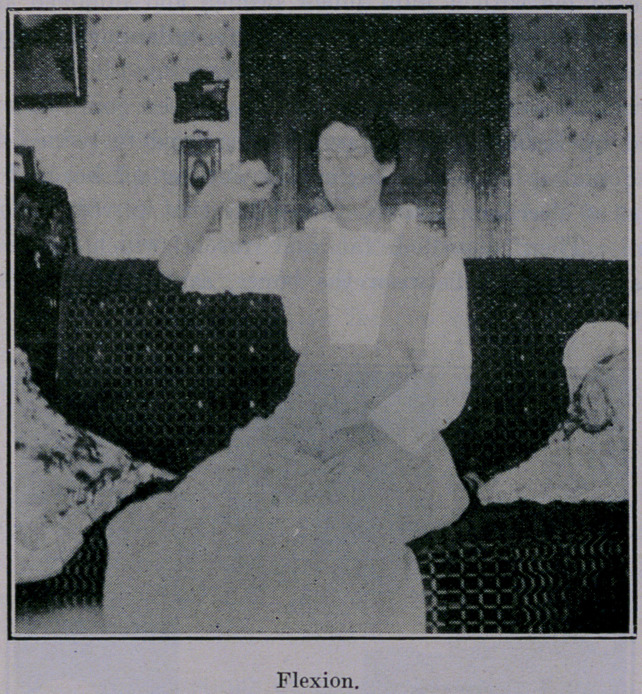


**Figure f3:**
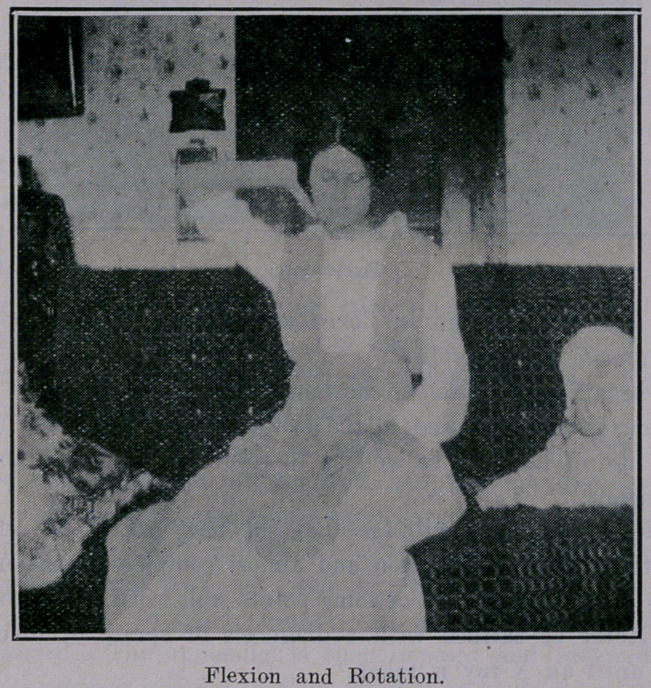


**Figure f4:**